# Teleosemantics, Structural Resemblance and Predictive Processing

**DOI:** 10.1007/s10670-024-00819-2

**Published:** 2024-07-01

**Authors:** Ross Pain, Stephen Francis Mann

**Affiliations:** 1 Department of Philosophy, Cotham House, University of Bristol, Bristol, BS6 6JL UK; 2 Department of Linguistic and Cultural Evolution, Max Planck Institute for Evolutionary Anthropology, Deutscher Pl. 6, 04103 Leipzig, Sachsen Germany

## Abstract

We propose a pluralist account of content for predictive processing systems. Our pluralism combines Millikan’s teleosemantics with existing structural resemblance accounts. The paper has two goals. First, we outline how a teleosemantic treatment of signal passing in predictive processing systems would work, and how it integrates with structural resemblance accounts. We show that the core explanatory motivations and conceptual machinery of teleosemantics and predictive processing mesh together well. Second, we argue this pluralist approach expands the range of empirical cases to which the predictive processing framework might be successfully applied. This is because our pluralism is *practice-oriented*. A range of different notions of content are used in the cognitive sciences to explain behaviour, and some of these cases look to employ teleosemantic notions. As a result, our pluralism gives predictive processing the scope to cover these cases.

## Philosophy, Cognitive Science and Representation

Philosophy and cognitive science have a complicated relationship when it comes to representation. Here is an illustrative caricature of that relationship. Cognitive science departments generate data, and attempt to explain that data using theories. Sometimes those theories posit representational content. At this point, philosophy departments sit up and take notice. Representational content is a long-contested notion in philosophy, and we can’t have other disciplines using it without proper analysis. Philosophers then assess how content could be attributed to cognitive systems in the context of the new theory. In a manner of speaking, then, philosophers *licence* the use of representational content.^1^

Predictive processing is a new, ambitious theory in the cognitive sciences. Proponents of the view treat the brain as a sophisticated hypothesis testing system. Models of the world are used to produce predictions of future sensory input, which are then updated based on any difference between predictions and actual sensory input (called *prediction error)*. This process results in more accurate predictions, which in turn means the system minimises prediction error over the long term (Clark, 2013; 2016; Friston & Kiebel, 2009; Hohwy, 2013). Linked probabilistic models of this sort are called “generative hierarchies” due to their ability to recreate incoming sensory states via top-down prediction (Hinton, 2007).

Advocates of the theory refer to “models of the world” (Hohwy, 2016, p. 281) being “encoded” and “updated” in the brain (Clark, 2017, p. 12) (Friston et al., 2011, p. 138) (Hohwy, 2016, p. 280) (Wiese & Metzinger, 2017, p. 10). It is also typical to speak of cognitive systems using these models to “compute predictions” (Clark, 2017, p. 9; Wiese & Metzinger, 2017, p. 5). A framework that appeals to encoded models of the world which compute predictions suggests an interpretation in terms of information-bearing structures that are produced, manipulated and stored by the brain. Consequently, it seems proponents of predictive processing will require a licence for representational content.^2^ In other words, we need some way of understanding how it might be that the various parts of a generative hierarchy come to be content-bearing.

Traditionally, it has been assumed that philosophy departments should issue *one type* of licence. This in turn has generated a lot of disputes among philosophers as they argue the case for their chosen account of content (Cummins, 1996; Dretske, 1981; Fodor, 1990; Millikan, 1984). Often, it is alignment with philosophical intuitions that guide these debates and constrains theory construction. But, as Shea succinctly puts it, “When it comes to subpersonal representations, it is unclear why intuitions about their content should be reliable at all" (Shea, 2018, p. 28). This suggests it is worth exploring other approaches to the problem. Another strategy, which has only gained interest more recently, acknowledges that finding one overriding account of representation for the cognitive sciences is unlikely to be successful. As such, philosophers should be sensitive to the fact that cognitive scientists employ a range of different notions of representation (Godfrey-Smith, 2004; Planer & Godfrey-Smith, 2021; Shea, 2018). We should hence be in the business of providing *pluralist* licences for content, precisely because the explanatory work facing cognitive science produces a range of different approaches to representation, which in turn require different notions of content. This involves a particular view on the role of philosophers of science in such debates, one which is more *sociologically*, or *practice* oriented (in what follows, we’ll use the latter term). The task facing philosophy is not to isolate a particular concept that covers all cases. Rather, it is to describe and clarify the range of different concepts that are used, or that might be used, to explain the workings of a successful scientific practice. Accordingly, philosophical intuitions do not play a central role in guiding theory construction in the practice-oriented approach.^3^ Our pluralism is motivated by this line of thinking.

To date, attempts to assign content to predictive processing architectures have appealed to *structural representations* (Gładziejewski, 2016; Kiefer & Hohwy, 2018; 2019). According to this view content is determined by a structural resemblance between an internal cognitive state and an external state of affairs. When applied to predictive processing, this is understood as the claim that the causal-probabilistic structure of generative hierarchies resemble the causal-probabilistic structure of the external world. We do not disagree with this approach; however, we think appealing to other theories of content, that have themselves been applied in cognitive science more broadly, can also be applied to predictive processing. Specifically, we appeal to teleosemantic thinking. This allows us to target a tightly specified sub-part of predictive processing machinery. Our approach is to outline how *signals* in generative hierarchies—that is, predictions and prediction errors—can be given a teleosemantic treatment. In what follows, we use Millikan’s sender-receiver model to argue that predictions represent external states of affairs and prediction errors represent the discrepancy between predictions and the states of affairs they predict. We thus advocate an account of the content-determining structures in predictive processing systems that appeals to both teleosemantics and structural representations. In other words, we issue a pluralist licence.

We have two main goals. Our primary goal is to show how a teleosemantic account of the content of signals in generative hierarchies would work. This takes up the majority of the paper. A secondary goal is to make the case for pluralism. We do not spend too much time on this task, as the fact that practice-oriented pluralism (as outlined above) is a position in the literature is reason enough to explore such treatments of predictive processing. Nonetheless, it is interesting to explore how pluralism plays out in this specific case. Predictive processing is claimed to be a highly general theory of action and perception, which applies to all cognitive systems (Hohwy, 2013; Clark, 2016). As such, it will need to be applicable across the phylogenetic spectrum. We think having teleosemantics on the table will help in this task. Accordingly, we expand on this motivation for our approach, and identify some specific cases where a pluralist treatment might be useful.

We proceed as follows. Section 2 provides a brief overview of predictive processing. Section 3 outlines Gładziejewski’s causal-probabilistic resemblance account of content in generative hierarchies. Section 4 provides a primer on teleosemantics. Section 5 gives our teleosemantic account of predictions and prediction errors. Section 6 makes the case for pluralism. Section 7 concludes.

## Predictive Processing

The literature on predictive processing is a large and complicated body of work, of which there are some excellent introductions (Clark, 2016; Hohwy, 2013). The overview we offer below is a general gloss, and is necessarily selective in the aspects it focuses on.^4^ In particular, we aim to draw out the sender-receiver structure of generative hierarchies in order to tie this with teleosemantic theory.

Our overview focuses on two features of the theory: (i) hierarchical prediction and prediction error; and (ii) prediction error minimisation.^5^ We address each in turn.

### Hierarchical Prediction and Prediction Error

**Fig. 1:**
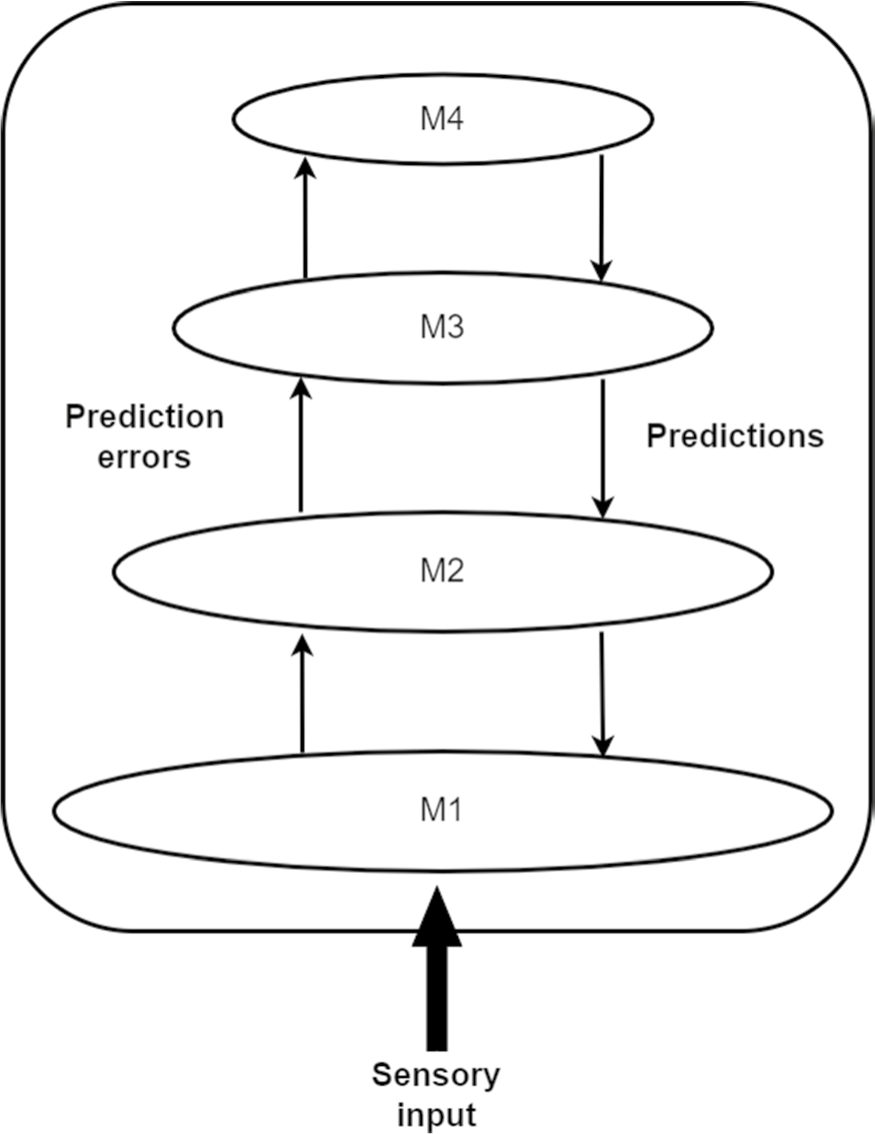
The mechanism at the core of predictive processing. Top-down transfer of predictions and bottom-up transfer of prediction errors across a hierarchy of models

The nature of bottom-up and top-down processing is re-conceived on the predictive processing framework. Top-down processing is understood in terms of prediction; more specifically, as *attempts to predict future sensory input*. Bottom-up processing is understood as the *transfer of prediction error*, where prediction error is the difference between predicted sensory input and actual sensory input (see Fig. 1).

Predictions are generated by encoded models of the world, which in turn are produced via experience, learning and evolution. These models incorporate hypotheses about the causes of sensory input, and generate predictions about future sensory input. They are hierarchically organised according to the spatiotemporal scales of the causal regularities they address. At lower levels in the hierarchy, models generate predictions at faster time scales and at more fine-grained spatial resolution; for instance, about which sensory transducers will be activated in the immediate future given those that are currently activated. At higher levels in the hierarchy, models generate predictions at slower time scales and at a broader level of spatial resolution; for instance, about the change in temperament of a friend after the birth of their first child. The predictions of models at the lowest level target the states of sensory transducers, whereas the predictions of any model above the lowest level target the states of the model directly below it.

Bottom-up processing is also reformulated on this account. Rather than being an encapsulated process in which perceptual experience is constructed from the raw data of sensory input, bottom-up processing is understood as the transfer of prediction error. At any given layer in the hierarchy, a model will receive prediction error signals from the model below it, attempt to explain away this error by refining its model, and forward any residual error that it cannot explain to the model above it.

### Prediction Error Minimisation

According to predictive processing, the central goal of a cognitive system is to minimise prediction error over the long-term. There are two ways in which the brain can deal with an active error signal. One option is to formulate a new hypothesis regarding the cause of the sensory input generating the prediction error. This can then be used to produce new predictions which can account for the error signal. On the predictive processing framework, this is the mechanism underlying perception, and is known as *perceptual inference*. Perception is understood as the product of the system’s ability to settle on a hypothesis that best explains sensory input; which is to say that prediction error is minimised. This process exhibits a mind-to-world direction of fit, in so far as states of the brain are adjusted in order to accommodate states of the world. Perceptual inference implies that, at every layer in the hierarchy, models are able to adjust their parameters according to the *content* of bottom-up prediction error signals. The content of these signals is, broadly speaking, the difference between (the content of) predicted sensory input and actual sensory input.

However, the brain also has the option of exploiting the world-to-mind direction of fit in minimising prediction error. In other words, it can adjust its place in the world in order to accommodate states of the brain. In this case the brain does not alter its hypotheses; instead it acts to bring about changes such that future sensory input matches the predictions of those hypotheses. On the predictive processing framework, this is the mechanism underlying action, and is known as *active inference*. More precisely, the brain generates action by predicting the proprioceptive sensory input given a hypothetical action, and then minimises the difference between its predicted sensory input and actual sensory input by changing the world or its position in the world. Importantly, active inference is recapitulated in the activity of each individual model in the hierarchy. Every model uses action—here the generation of predictions—to influence the states of the model below it in ways that will alter incoming prediction error, and hence the sensory states of the original model. That is, each model uses its active states to influence its sensory states. This top-down influence of higher models on lower models is typically described in terms of “modulation" or “guidance" (Clark, 2016, p. 146; Kirchhoff et al., 2018).

So, according to predictive processing, both perception and action are products of the more general imperative to minimise prediction error, and hence are explained by appeal to a single computational mechanism. Moreover, the theory implies that every model in the hierarchy is able to *produce* contentful predictions and prediction errors, and is in turn capable of adjusting its parameters *in response to* contentful predictions and prediction errors. This part of the predictive processing mechanism will be the target of our teleosemantic analysis.^6^

### The Sinister Figure Example

A simple example (one that will be familiar to most) illustrates the mechanism being proposed here. Imagine that you have just woken up in the middle of the night. As you yawn and stretch, you happen to glance toward the corner of your room, and see what looks to be a sinister figure lurking there. Startled, you quickly sit up and turn on the light. Thank God, you gasp—it was just a pile of clothes strewn across a chair!

According to predictive processing, this case should be analysed as follows. The hypothesis that the cause of your initial sensory input was a (sinister) figure provides an excellent explanation of that input. As such, the best way to minimise prediction error was to deploy the sinister figure hypothesis; which in turn explains the character of your visual experience. But the alarming nature of that experience immediately brings about the need to investigate further. The sinister figure hypothesis generates the prediction that you will get a better look at whoever it might be if you sit up and turn on the light. This high-level prediction modulates the behaviour of models below it, which in turn produce further predictions, and so forth down the hierarchy. A cascade of predictions relating to the hypothetical action—you turning on the light—are thus generated. If the hypothesis ‘I am turning on the light’ is held fixed, this will result in a corresponding cascade of prediction errors rising up the hierarchy, as sensory input will not match predictions. By moving in such a way as to turn on the light, this error signal is minimised. However, the new sensory state generated by turning on the light is not explained by the original (sinister figure) hypothesis. So again we have a difference between predicted sensory input and actual sensory input. Consequently, a new hypothesis must be deployed to suppress the error rising through the system. The hypothesis that there is a pile of clothes on a chair in your room explains the new sensory input well. By producing a new hypothesis–the untidy chair hypothesis–the error signal can be explained away. Prediction error is then minimised if the system settles on this hypothesis.

Although just a toy example, this gives us an idea of how predictive processing understands the computational link between action and perception. In the end, both are strategies the brain uses to minimise prediction error. Furthermore, the combined processes of predicting sensory input and updating models in response to prediction error allow the system to build increasingly accurate models of the world. A cornerstone of the framework is that every model in the hierarchy is able to produce and respond to contentful predictions and prediction errors.

The preceding discussion raises two important questions. First, in what sense do models become ‘increasingly accurate’? Second, how do prediction and prediction error signals get their content? In the next three sections we address these questions.

## A Structural Resemblance Account of Content for Generative Hierarchies

We noted in the introduction that previous attempts at ascribing content to predictive processing architectures have appealed to structural resemblance. We agree that this strategy constitutes a plausible theory of content for generative hierarchies. In this section, following Gładziejewski (2016), we outline the sense in which internal models structurally resemble the external world. In the following sections, we outline a teleosemantic theory of the content of signals in predictive processing architectures.

The core claim put forward by proponents of structural representations is that content is determined, to some extent, by a structural resemblance between an internal cognitive state and an external state of affairs. The challenge is then to determine precisely what this structural resemblance amounts to, in any particular case of representation. Gładziejewski (2016, p. 566) cites cartographic maps as the “golden standard" for structural representations. This is because: (1) they are representational; (2) they guide the actions of their users; (3) they do so in a detachable way; and (4) they allow their users to detect representational errors. Fulfilling the latter three conditions is an important part of any theory of representation (especially if, following Gładziejewski, we want to meet Ramsey’s (2007) job description challenge). However, here we will focus on the first condition: how exactly is it that models in predictive processing architectures structurally resemble external states of affairs?

When it comes to cartographic maps, the structural resemblance relation is *spatial*. For example, if my map of the university depicts the cognitive science department as being closer to the cricket pitch than the philosophy department, then we can conclude that the layout of the university itself is such that cognitive science department is closer to the cricket pitch than the philosophy department. Of course, in the case of predictive models, it is implausible that the structural resemblance relation is between spatial quantities. Rather, the claim is that the *causal-probabilistic* structure of internal models resembles the *causal-probabilistic* structure of external states of affairs.

Gładziejewski (2016, pp. 571–572) argues that causal-probabilistic resemblance has three dimensions. The first of these is a probability distribution, which defines a *likelihood*. According to predictive processing, variables in a model encode the probability of some sensory input occurring given some external state of affairs.^7^ The claim, then, is that the relation between variables in a model and lower-level sensory activity structurally resembles the relationship between worldly causes of that sensory activity and the activity itself. For an example we will repeatedly draw on below, consider the capacity of a trained rat to press a lever to retrieve food. The rat’s hierarchical model represents the lever in terms of the probability that certain sensory patterns are produced; from short-term time scales—such as the colour and shape of the lever—to more long-term time scales—such as the interoceptive sensations associated with the digestion of food. The probabilistic relationship between the lever-representing model and sensory input thus structurally resembles the causal relationship between the actual lever and sensory input.

However, models do not predict sensory input in a straightforward manner. As we have seen, the system as a whole predicts sensory input transitively, in that higher-level models produce predictions of activity in lower-level models. This suggests a causal-probabilistic structural resemblance between (on the one hand) the values of interacting variables evolving via inter-model dynamics and (on the other) causal relationships between objects in the world. If, for example, there is a causal relationship between lever-pressing and food, then this relationship should be recapitulated in the way that the values of different variables across models influence one another. So levers can be represented not only in terms of their relationship to future sensory input, but also in the way they causally interact with other objects. This is the second dimension of structural resemblance.

Models also structurally resemble causal-probabilistic relationships in the world via encoded priors. If a generative hierarchy is to realise Bayesian reasoning, it must be capable of comparing the probability that a lever is the cause of current sensory input with the probability that the system would encounter a lever, *independently* of the evidence provided by current sensory input. For instance, if it is more likely that our trained rat encounters actual functioning levers, rather than objects that look like levers but cannot be pressed, then the system should prefer the former hypothesis. The values of priors thus structurally resemble the *experience-independent* causal-probabilistic structure of the world. This is the third dimension of structural resemblance.

We now have a sketch of how content in generative hierarchies might be understood in terms of causal-probabilistic resemblance with the world. However, given our practice-oriented approach, it will be useful to have more than one account of content on the table. This will allow predictive processing to be applied in case studies that might require different notions of content. In Sect. 5, we will outline how teleosemantics can provide an account of content for signal passing between models. In Sect. 6, we explain why this is important and describe such a case study. But first we offer a brief primer on teleosemantic theory.

## Teleosemantics

Teleosemantics defines a representation as an intermediary between two cooperating devices: (1) a sender, which produces the intermediary, and (2) a receiver, which conditions its behaviour on the intermediary.^8^ The sense in which these devices must be ‘cooperating’ is cashed out in terms of *proper functions*. A proper function is a causally downstream outcome that a device has been selected for bringing about, either through natural selection, reinforcement learning, explicit design or some other appropriate selection process.^9^

We will briefly introduce proper functions before describing their role in the definition of representational content. Many biological devices are adaptations, having selected effects that contribute to their proliferation. The mammalian heart, for example, has a selected effect to pump oxygenated blood around the body. In achieving this effect hearts contribute to the reproduction of the genes that produced them, thereby contributing to the production of more hearts in future. When causal effects lead devices to be reproduced, teleosemantics calls those effects proper functions. However, the term is not only applied to devices produced by genes proliferating due to natural selection. Any device that owes its present form to selection on the effects of its ‘ancestors’ has a proper function. Consider again the capacity of a trained rat to press a lever to retrieve food. This capacity has lever-pressing as a proper function. A lever-pressing disposition has been reinforced by the reliable appearance of food after individual lever-pressing events. The disposition ‘proliferates’ because previous manifestations of that disposition were followed by consumption of food. For a disposition to proliferate here means being more likely to occur in a given environment than other possible dispositions. Reinforcement is therefore construed as selection (Hull et al., 2001); it is differential retention of a certain disposition (lever-pressing) and is relevantly similar to the kind of process exemplified by natural selection. In the case of reinforcement learning, the ‘ancestors’ of a present behaviour are earlier instances of that disposition performed by the learner.

**Fig. 2:**
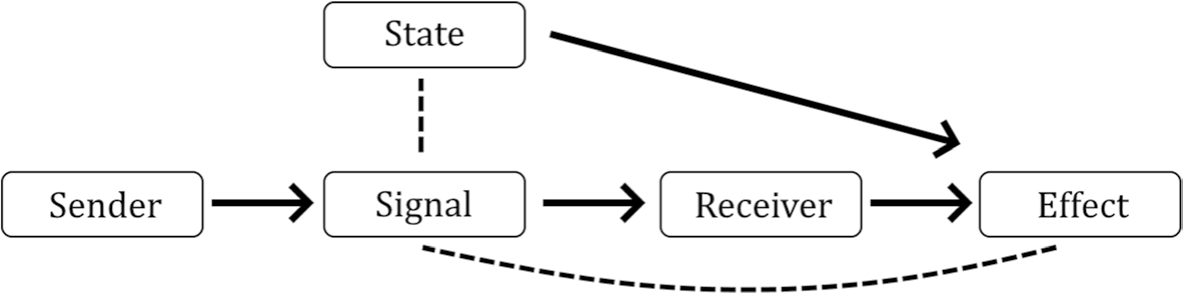
The basic teleosemantic model. The **Receiver** has a proper function to bring about some **Effect** (in a causal model, this function would be specified as a requirement to set the effect variable to a certain value). However, the receiver is hindered by interference from some **State**, causally upstream of the effect, on which the receiver *cannot* directly condition its behaviour. The **Sender**, which has a proper function to help the receiver achieve its function, produces a **Signal** on which the receiver *can* condition its behaviour. Teleosemantics asserts that when the receiver conditions its behaviour on the signal and is more successful than it would have been otherwise, this increased success can only be fully explained by adverting to a relation between the signal and the state. This relation is then the basic representational relation, or descriptive relation. The signal bears a directive relation to the proper functional effect (descriptive and directive relations illustrated with dashed lines). This figure and caption first appeared in Mann & Pain (2022).

How do proper functions generate representational content? Entities that stand in a sender-receiver relationship to each other, and have a shared proper function as a consequence of selection, endow their intermediaries with representational content. The justification for this definition is as follows. The shared proper function is a downstream causal effect that the receiver must exercise causal influence to bring about, modelled in Fig. 2 as a certain value of the ‘Effect’ variable. However, external states of the world also have causal influence on the effect, meaning the receiver cannot simply act to produce the desired value. If the receiver could condition its behaviour on the external state, it could produce an appropriate act in order to ensure the effect takes the value required. But it cannot observe the state directly: the best it can do is condition its behaviour on the intermediary. When conditioning on the intermediary leads to greater success than acting unconditionally, teleosemantics asserts that this must be due to a relation between the intermediary and the external state. Teleosemantics identifies this relation as the basic form of representational content.^10^ When these circumstances hold, the intermediary is a representation and the external state is its truth condition.

There are in fact two kinds of basic representational relation. The one more commonly referred to is the *descriptive relation*, which holds between the signal and the external state. The other is the *directive relation*, which holds between the signal and the proper functional effect it is supposed to help bring about. Because teleosemantics was originally developed as a theory of human natural language, the two basic relations are usually associated with indicative sentences (that say how the world is) and imperative sentences (that say what action to take). In basic systems, these two aspects are tightly coupled. A signal will have one particular state to which it corresponds, and simultaneously one particular act it is supposed to prompt. In more complex systems, descriptive and directive aspects can come apart. There can be purely descriptive signals, which correspond to individual states of the world but do not prompt any single action. Complex systems can combine descriptive signals to form an accurate picture of the world and guide flexible behaviour. There can also be purely directive signals, which prompt specific actions but need not be tied to specific environmental circumstances.

The basic teleosemantic framework depicted in Fig. 2 occurs within models of cognition, and practitioners often draw on concepts of signalling, messaging, information or representation in giving explanations. The theory thus offers an attractive option for understanding the content of prediction and prediction error signals in generative hierarchies, especially within the context of the practice-oriented approach.

## A Teleosemantic Account of Content for Predictions and Prediction Errors

In this section we bring together teleosemantics, predictive processing, and structural resemblance. Our goal is to show how predictions and prediction error signals get their content.

### Models in the Hierarchy are Senders and Receivers

Predictions and prediction errors are signals sent between models in the generative hierarchy. Models play the role of senders and receivers in the teleosemantic framework. Consequently, our initial task is to address the following question: what is the proper function of a model in a generative hierarchy? At first pass, there look to be at least two plausible answers to this question.

In the broadest sense, a model is adaptive in so far as it is accurate with respect to the world. As we have seen, on Gładziejewski’s structural resemblance account, models resemble the causal-probabilistic structure of the world. To increase a model’s accuracy is thus to increase its causal-probabilistic resemblance with the world. All other things being equal, this allows an organism to interact more successfully with its environment. For instance, in the case of a trained rat, an accurate model will more reliably bring about the pressing of a lever that delivers food. So we might want to say that, in general, the proper function of a model is to accurately represent the world.

However, a model does not have direct access to the world; how then can it accurately represent it? In the case of our rat, the problem is that the success-relevant effect—that is, the pressing of the lever—requires having an accurate model of a state of the world—that is, the lever itself. But the model cannot directly condition its behaviour on that state. What the model *can* directly access is the incoming sensory signal, and the flow of top-down predictions and bottom-up error signals. As we have seen, a core commitment of predictive processing is that by conditioning their behaviour on these signals, models will become more accurate with respect to the world. Hohwy (2013, pp. 50–51) argues that, for a model in a predictive processing hierarchy, increasing mutual information with worldly affairs is extensionally equivalent to minimising prediction error. On the structural resemblance account outlined above, a model increasing its mutual information means that the values of hidden variables will come to map more reliably on to causal-probabilistic relationships between objects in the world and an organism’s sensory states. Consequently, in a more restricted sense, we can say that models are adaptive in so far as they minimise prediction error. It is hence possible to understand prediction error minimisation as the proper function of a model.

The upshot is this. Minimally, the proper function of a model is to minimise prediction error. However, given this entails that mutual information between a model and the world is maximised, this is extensionally equivalent to saying that the proper function of a model is to accurately represent the world. And in any specific case, this will cash-out as the need to accurately represent some particular part of the world. For instance, an accurate model of a lever is selected for in a rat via learning because it aids in the pressing of the lever, which delivers food.

The core commitments of teleosemantics and predictive processing thus mesh together well. Predictive processing offers a mechanism for understanding how the brain overcomes the central inferential problem it faces: identifying the external structure of the world from the noisy, uncertain signals it has direct access to. The structure of this mechanism should be familiar to teleosemanticists: by conditioning its behaviour on an internal signal, a device can aid an organism by producing adaptive responses to the external environment. What teleosemantics offers is a way of understanding *why* predictions and prediction error signals can be understood as representational. This is because explaining the increased success produced by more accurate models requires positing a relation between intermediaries—predictions and prediction errors—and external success-relevant circumstances. In the remainder of this section, we run through the mechanics of this proposal in more detail.

### The Content of Prediction Signals

**Fig. 3:**
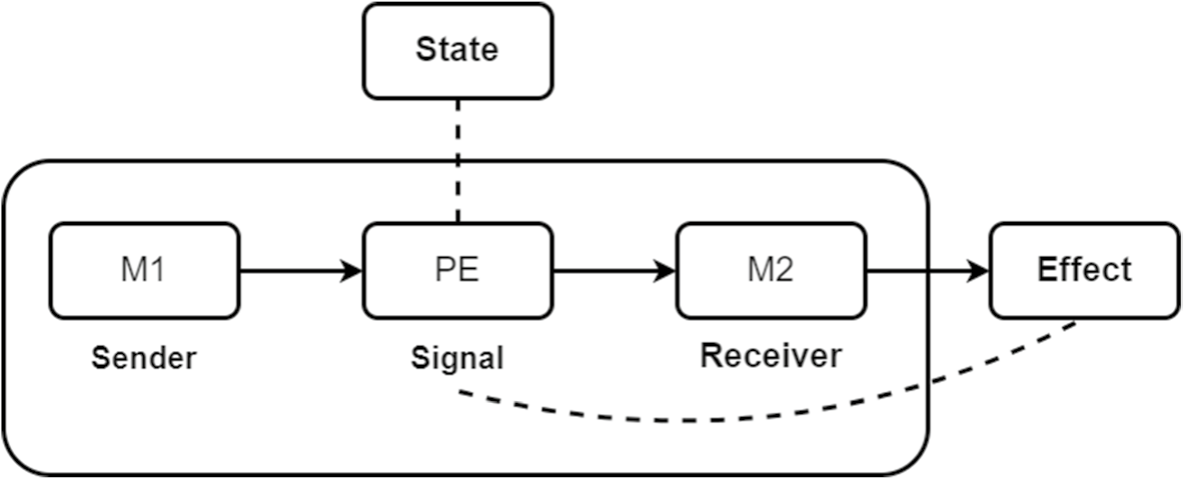
The content of prediction signals. **P**: Prediction; **M1**: a lower model in the hierarchy; **M2**: a higher model in the hierarchy. **M2** emits **P**, which determines the priors of **M1**. These quantities are then held fixed, such that minimising the error raised against them results in bringing about the effect that is the proper function of **M1**. Over the long term, this process will both increase mutual information between models and the world and increase the accuracy of the system’s predictions. According to teleosemantics, explaining this success requires positing a relation between **P** and the external success-relevant circumstances. A descriptive relation (represented with a dashed line) holds between **P** and upcoming sensory input of **M1**. In the case of active inference, the content of **P** will *mis*-represent some state of the world. A directive relation (represented with a dashed line) holds between **P** and the effect that it is the proper function of **M1** to bring about: altering the priors that encode its expectations about future sensory input, and eventually raising a prediction error if that input diverges from **P**

According to predictive processing every model throughout the generative hierarchy is constantly issuing predictions about the sensory input of the model directly below it. More specifically, higher models in the hierarchy issue predictions of future sensory input which determine prior distributions used by lower models. When these predictions fail to match the sensory input the lower model receives from even further down, error begins to rise in the system. By adjusting states of the world and their place in it, organisms can reduce this error. From a teleosemantic perspective we can understand the higher model in the hierarchy as the sender, the lower model as the receiver, and the prediction as the signal (see Fig. 3).

The two models are a pair of cooperating devices. The proper function of the receiver-model is to minimise prediction error over the long term and thus maximise its accuracy with respect to the causal-probabilistic structure of the world. But attaining these success conditions involves tracking circumstances that the model cannot directly access (long-term error minimisation and states of the world). The sender-model emits a prediction signal, on which the receiver-model conditions its behaviour. More specifically, the prediction signal modulates the priors of the receiver-model, such that they reflect (at a finer spatio-temporal grain) the priors of the sender-model. The organism will then act to reduce the error that arises from the predictions produced when model priors are set in this way. This process of actively testing predictions against the world minimises prediction error over the long term. Consequently, by conditioning its behaviour on the prediction signal, the receiver-model is better able to achieve its proper function.

On the teleosemantic analysis, explaining this success requires positing a relation between the internal signal and an external success-relevant condition. In the case of our trained rat, successful active inference will more reliably bring about lever-pressing. The goal of lever pressing is selected at the highest level in the rat’s cognitive system. Each model in the system then modulates its priors according to top-down predictions regarding the sensory input expected from pressing the lever. The priors of the models are held fixed, and hence the only way to reduce the ensuing prediction error rising up through the system is to move in such a way as to match the initial predictions. This then brings about the actions required to complete the goal of lever-pressing. There is hence a descriptive relation between the prediction signal and the lever. In the case of active inference, initially this descriptive relation will *mis*-represent the lever. That is, it will predict the sensory input associated with the pressed lever, and not as the lever currently is (unpressed).^11^ The prediction signal will come to accurately represent lever-pressing when the motor system has moved the body in such a way as to reduce error and bring about the system’s goal. Thus there is a directive relation between the prediction and the external effect of lever-pressing.

The portrayal of action as a form of inference highlights a clash of perspectives between active inference and teleosemantics. Proponents of active inference say that since the process by which actions are chosen is relevantly similar to the process by which models are updated, we should describe action as a form of inference. Contrariwise, proponents of teleosemantics say that since anything that plays the role of action in the teleosemantic schema counts as action, and updating a model counts as action in the schema, so perceptual inference (which consists in updating a model) counts as action. We believe this is a difference of perspective rather than a disagreement over matters of fact.

### The Content of Prediction Error Signals

**Fig. 4:**
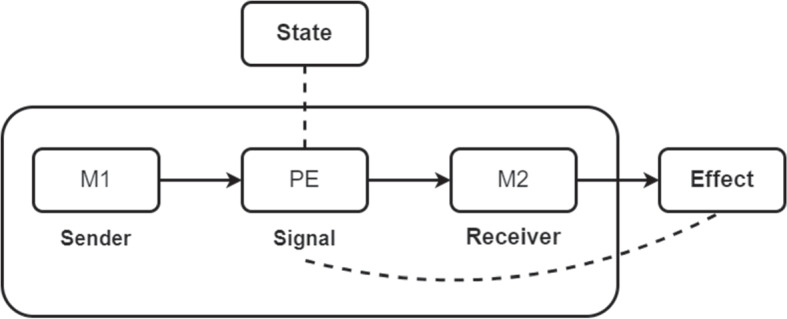
The content of prediction error signals. **PE**: Prediction Error; **M1**: a lower model in the hierarchy; **M2**: a higher model in the hierarchy. **M1** emits **PE**, on which **M2** updates its priors in order to account for the error. Conditioning its behaviour in this way will both increase mutual information between itself and the world and increase the accuracy of the model’s predictions. According to teleosemantics, explaining this success requires positing a relation between **PE** and the external success-relevant circumstances. A descriptive relation (represented with a dashed line) holds between **PE** and the magnitude of the difference between earlier predictions of **M2** and sensory input received by **M1**. Because it concerns the content of the original prediction signal, the prediction error signal is a metarepresentation. A directive relation (represented with a dashed line) holds between **PE** and the effects that it is the proper function of **M2** to bring about: either updating its priors (inference), or effecting some change in the world (action); either of which should serve to quash future prediction errors

Recall that on the predictive processing story, bottom-up processing involves the transfer of prediction error. More specifically, each model in the hierarchy receives error signals from the one below it, adjusts its priors in an attempt to account for the error, and forwards any residual error to the model above it. This is the mechanism of perceptual inference. From a teleosemantic perspective we can treat the lower model in the hierarchy as the sender, the higher model as the receiver, and the prediction error as the signal (see Fig. 4).

The two models are a pair of co-adapted, cooperating devices. The proper function of the receiver-model is to minimise prediction error over the long term and thus maximise its accuracy with respect to the causal-probabilistic structure of the world. But attaining these success conditions involves tracking circumstances that the model cannot directly access (long-term error minimisation and states of the world). The sender-model emits an error signal, on which the receiver-model conditions its behaviour. More specifically, the receiver-model will update its parameters in an attempt to account for the incoming error signal. If this process is successful the model increases its accuracy, which has the effect of producing more accurate predictions in the future and hence minimises prediction error over the long term.

**Fig. 5:**
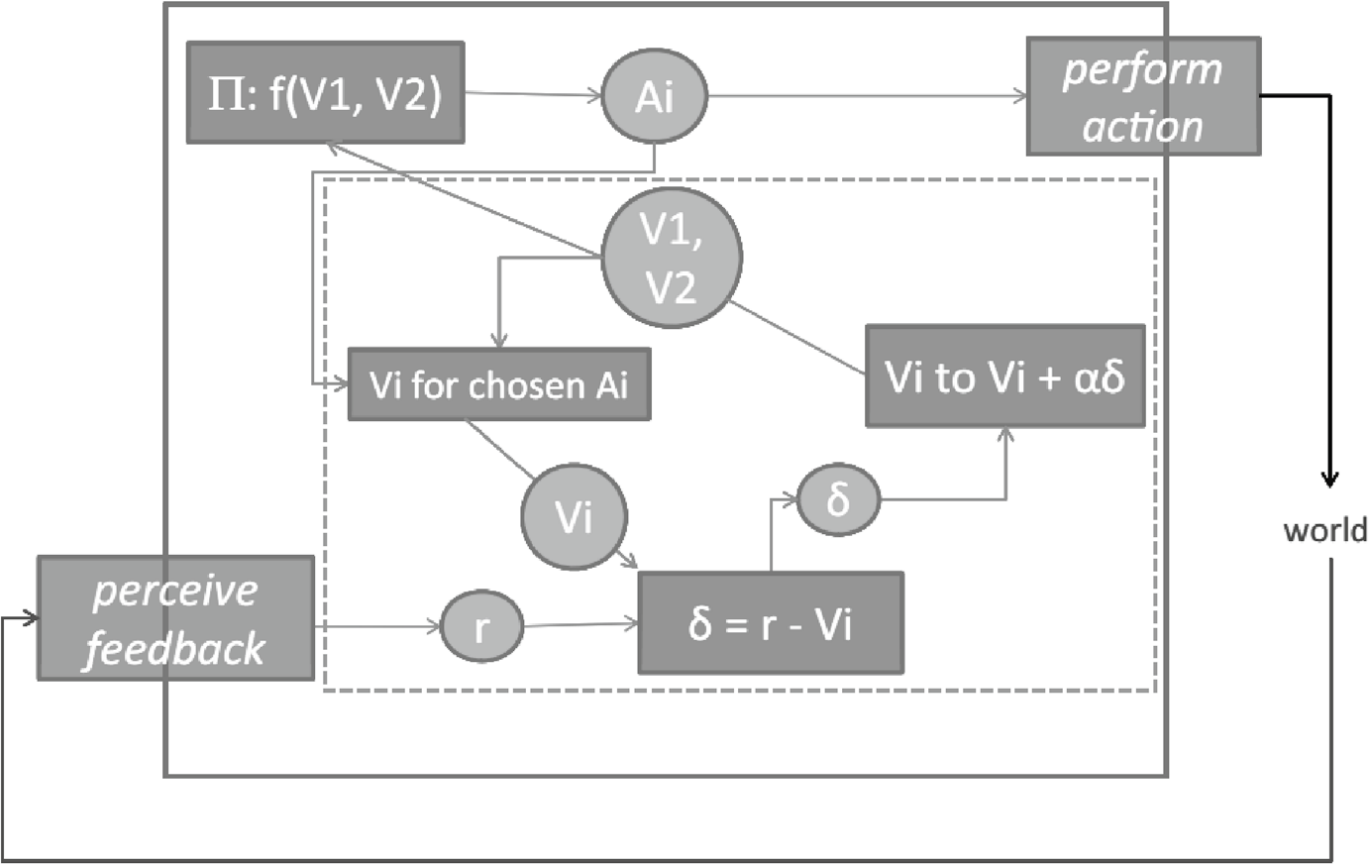
Simplified form of the actor-critic framework discussed by Shea (2014, p. 320, Fig. 1). The system employs a decision procedure $\Pi$ that chooses acts $A_i$ in proportion to their expected payoffs $V_i$. The actual payoff, ***r***, of an act at the previous timestep is used to update the system’s estimates of $V_i$. This is done by generating a prediction error signal indicating the magnitude of the difference, $\delta$, between the expected reward and the actual reward. The system’s representation of the expected reward is updated based on this error and a learning parameter $\alpha$. We have added a dashed-line box picking out the subsystem that can be generalised to a model-to-model relationship within a generative hierarchy (Fig. 6)

Prediction errors appear to be metarepresentational. Their content concerns the content of predictions, in that they say whether and how much a prediction was inaccurate. Shea (2014) has argued that a particular class of signals in the brain, bearing some similarities to the prediction errors discussed here, are metarepresentational. The context of the argument is a particular computational model of neural processing, the actor-critic framework, within which a reward prediction error signal appears (Fig. 5).^12^ Shea argues that error signals in this framework are metarepresentational, with their contents being about the inaccuracy of another (first-order) representation.

It is worth seeing whether Shea’s account applies to prediction error signals in the predictive processing hierarchy, and so it is worth outlining similarities and differences between the hierarchy and the actor-critic framework on which Shea’s account is based. First, Shea is making claims about specific signals that have been discovered in the brain. Computational cognitive scientists have established that the actor-critic framework is a good way to understand the dynamics and function of this part of the brain, and so the prediction error signals that appear in that framework are appropriately identified with the brain signals that play the equivalent prediction error role. We by contrast are discussing hypothetical prediction error signals that would be found in the brain if the generative hierarchy turns out to be an accurate depiction of brain activity. We don’t regard it as settled that the brain contains generative hierarchies but, if it does, we are committed to the claim that the contents of prediction errors are as we describe them here. Second, the actor-critic framework is much simpler than the predictive processing framework. The computations carried out by an actor-critic system are called *model-free*, in that there is no component representing causal relationships. There is just a point estimate representing the expected reward for a particular behaviour. It is this point estimate whose inaccuracy the prediction error signal indicates. By contrast, the predictive processing hierarchy is decidedly not model-free: it contains models whose purpose is to represent causal-probabilistic features. So the first-order representation whose content the prediction error signal indicates cannot be exactly the same component in the actor-critic framework and in the predictive processing framework. Instead, the prediction error indicates the inaccuracy of the prediction itself, not the model that emitted the prediction.

**Fig. 6:**
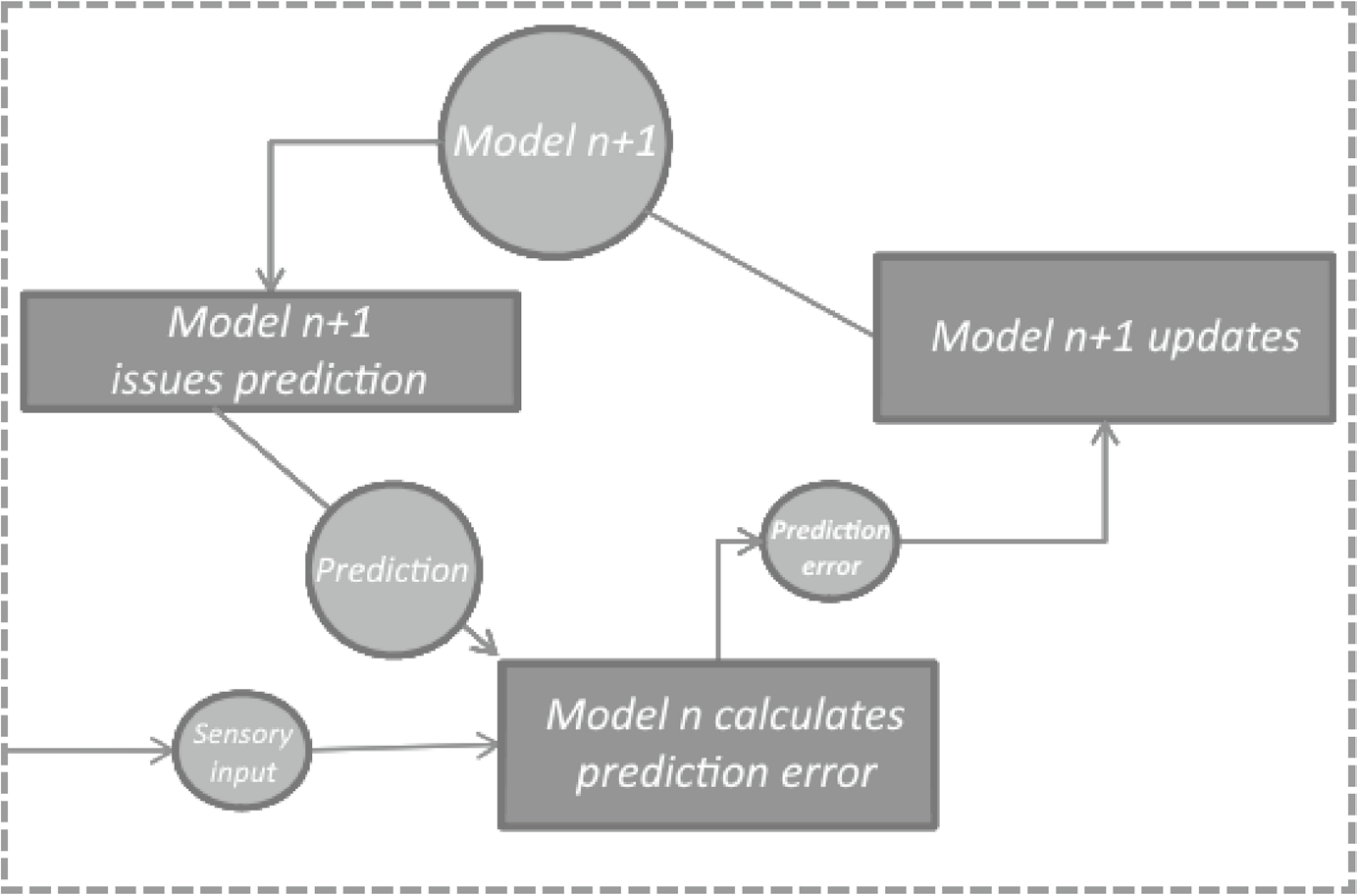
The boxed portion of the actor-critic framework (Fig. 5) is a degenerate kind of predictive processing architecture. The main text leverages Shea’s argument to establish the claim that prediction error signals have metarepresentational content. Note that the component types in this figure do not match component types in the generative hierarchy, because the actor-critic framework is a ‘model-free’ means of using feedback to update representations. That is why the model at level $n+1$ here appears in a circle, while the model at level *n* appears inside a rectangle: the actor-critic framework is cast in terms of representations and linear operations, rather than models and signals

Although the first-order representation whose content the prediction error signal concerns is the prediction rather than the model that emitted it, a version of Shea’s argument in favour of metarepresentational content still goes through. Prima facie, the prediction error signal is metarepresentational. Its content is that the prediction was accurate or inaccurate. The content of the prediction error signal is that the prediction was in error by such-and-such an amount. It is this metarepresentational content that explains why the model updates its priors; when the signal correctly indicates the error in the prediction, the model’s updates cause it to produce more accurate predictions in future. In a way, this is a more general case of the actor-critic framework (Fig. 6). In the actor-critic framework, the system keeps track of just one feature of the external world (the expected reward) and emits just one kind of prediction (also the expected reward). In the predictive processing framework, a model keeps track of multiple features of the external world (every causal-probabilistic relationship that model represents) and emits multiple kinds of prediction (anything the creature could encounter that it is that particular model’s job to keep track of; i.e. anything at the appropriate level of spatiotemporal grain). Predictive processing systems are multi-tasking actor-critic systems. If we accept Shea’s claim of metarepresentational content in the latter, there is no special reason to withhold it from the former.

By conditioning its behaviour on the error signal, the receiver-model is better able to achieve its proper function. As we have seen, according to teleosemantics explaining this success requires positing a relation between the internal signal and an external success-relevant condition. Take the case of a model in a rat’s cognitive system whose proper function is to aid lever-pressing. The model adjusts its priors according to the bottom-up error signal. The proper function of the model determines the correspondence the error signal bears to the lever. Importantly, the general content of an error signal will always be the difference between predicted sensory input and actual sensory input. And in this particular case, the content will be the difference between the prediction initially issued by the model regarding expected sensory input caused by the lever and actual sensory input caused by the lever. There is hence a descriptive mapping relation between the prediction error signal and the lever, and a directive mapping relation between the prediction error signal and the external effect of lever-pressing.

### The Sinister Figure Example: Teleosemantics Version

Let’s now run the sinister figure example through our hybrid structural resemblance-teleosemantic account. Initially, when you wake, the sinister figure hypothesis dominates. Prediction error is minimised if that hypothesis is deployed, as it best explains your current sensory input. Models in the system adjust their priors and issue predictions accordingly. Both predictions and prediction errors bear a descriptive relation to the untidy chair, with the indicative content <there is a sinister figure>. Of course, here that content is *inaccurate* with respect to the world.^13^ The sinister figure hypothesis also allows the system to raise new predictions, such as the prediction that turning on the light will reveal the identity of the sinister figure. This will produce corresponding prediction error, which can be minimised if you act in such a way as to bring the prediction about. Predictions (and hence prediction errors) bear a directive relation to the external state of affairs of turning on the light, with the imperative content <turn on light>. Here the system exploits a world-to-mind direction of fit. However, in this case the outcome of turning on the light will generate a mismatch between predicted sensory input and actual sensory input. In order to eliminate this error, a new hypothesis will be raised: the untidy chair hypothesis. Here the system exploits a mind-to-world direction of fit. The fact that models in the system condition their behaviour on the error signal here indicates that there is a representational relation between the error signal and the success-relevant external circumstances; that is, the untidy chair. The new hypothesis produces predictions bearing a descriptive relation to the untidy chair, with the indicative content <there is an untidy chair>.

This illustrates the neat way in which predictive processing and teleosemantics mesh. By minimising error, predictive brains are able to increase the accuracy of their models, despite having no direct link to the causes of their sensory inputs. Via appeal to success-relevant circumstances, teleosemantics gives us an account of how the flow of predictions and error can bear content about the external world; again, despite the brain having no direct contact with those circumstances.^14^ The overall picture we are advocating is that generative hierarchies are able to increase their structural resemblance with the world by processing signals with teleosemantic content.

### Two Objections

We now consider two important objections to our account.^15^ The first is that it seems wrong to treat higher-level models as senders and lower-level models as receivers. The second is that it seems wrong to treat the content of a first-order representation (i.e. a model) as dependent on the content of a meta-representation (i.e. an error signal). We address each in turn.

Intuitively, it seems strange to assign the role of sender to a higher-level model and the role of receiver to a lower-level model. Higher models lie ‘deeper’ within the cognitive system, further from the sensory surface and thus further from the world which they are supposed to be providing information about. Signals are supposed to provide information about external states of affairs. But how can a model that is physically further away from the world provide a model that is physically closer to the world with information *about the world*? By contrast, the usual way the sender-receiver framework is applied to cognitive systems treats sensory apparatus as the sender and motor apparatus as the receiver; this makes sense because sensory apparatus has access to worldly information that motor apparatus does not. Our application of the framework to the predictive processing hierarchy seems to get things the wrong way round.

To respond, our application of the sender-receiver framework makes sense when we consider the different information that is stored in models at different levels. Higher models store information that is relevant on longer timescales or that concerns objects and events that are more causally opaque. It is true that they build up this information from the signals that are passed to them from the lower levels. But it need not be true that the predictions they pass back down the hierarchy contain information that those lower levels already possess. For one thing, there could be multiple lower models serving a single higher model, such that the higher model is able to integrate information and generate predictions that no single lower model could have access to. For another, the lower models might simply fail to encode and store information that is nonetheless transmitted further up the hierarchy, such that it is news to them when it comes back in the form of predictions. Consider by way of analogy a housebound analyst who receives letters from servants gathering information from the outside world. If the servants were numerous enough and forgetful enough, eventually the analyst could gather more information (and issue more accurate predictions) than any single servant.^16^

The second objection stems from our characterisation of prediction error signals as metarepresentational. Our picture seems to suggest that the accuracy of a first-order representation (i.e. a model in the hierarchy) is made possible by a metarepresentation (i.e. an error signal). This looks problematic: presumably metarepresentations cannot be prior to the first-order representations they metarepresent. We should instead tell a story on which first-order representations come first and metarepresentations are defined subsequently.

To respond, first note that Shea’s account has the same consequence. We characterised predictive processing hierarchies as multi-tasking actor-critic systems, and in both cases a first-order representation is kept attuned to the world by use of an error signal. The use of an error signal to improve the accuracy of a first-order representation does not threaten its status as first-order. There is a difference between how the first-order representation gets its content and how it is kept accurate. So if we can give an account of how the first-order representation gets its content independent of any metarepresentational updating, we will have avoided the problem. And our account is just that the content of a model derives from its structural resemblance with external affairs. A model is a structural-resemblance representation that does not depend on error signals for its representational status or for its content, though it does utilise error signals to improve its accuracy. One might wonder how a model can gain representational status before the predictive processing hierarchy is ‘brought to life’, so to speak, with its first bouts of signalling. One possibility is to appeal to innate priors, such that a hierarchy has some amount of in-built structure that very loosely tracks (i.e. structurally resembles) features of the world. Brains are imbued with these in-built first-order representations, that may be vague or inaccurate at the outset, and are then iteratively updated through experience. This is one possible way in which models can be attributed first-order representational content before the predictive processing hierarchy kicks into life; there may be others. The important point is that first-order representations do not depend on metarepresentations for their content or representational status, even if they do depend on them to remain accurate.

## Why We Should Issue Pluralist Licences

We have offered a pluralist account of content for predictive processing architectures: models in generative hierarchies get content in virtue of their causal-probabilistic resemblance with the world; while signals get their content in virtue of their etiology. In this section we explore in more detail the motivating reasons for adopting a practice-oriented pluralism.

### Practice-Oriented Pluralism

Some may worry about pluralism. Shouldn’t we want to give a single overarching account of content in predictive processing architectures? Isn’t a unified account preferable to meshing together two different accounts? After all, the claim that content is determined by histories of selection and the claim that content is determined by structural resemblance are very different claims: why think they will play nicely together? Methodological pluralism is not always a good thing, especially if you inherit the problems of both theories.

We think there are good reasons to adopt a pluralist approach to cognitive representations despite these concerns. Here we align with those who express pessimism at the chances of ever finding a single unifying theory of representation via philosophical means alone. Although the prospects for such a theory looked promising in the 1980s—particularly through the work of Fodor, Dretske and Millikan—problems persist.^17^ As a result, many feel those projects failed to deliver (Godfrey-Smith, 2004; see also Planer & Godfrey-Smith, 2021; Shea et al., 2017). One reason for this is that cognitive science spans the domains of folk-psychology and scientific-psychology. This requires—to borrow Wilfrid Sellars’ famous terms—going back and forth between the manifest and scientific images. Given such disciplinary complexity, we should expect to see a diversity of accounts of content emerge. Peter Godfrey-Smith puts the point as follows:

Cognitive scientists forge different kinds of hybrid semantic concepts in different circumstances—in response to different theoretical needs, and different ways in which scientific concepts of specificity and folk habits of interpretation interact with each other.

Godfrey-Smith (2004, p. 160)

Given this situation, what is the role of philosophers of cognitive science working on content? One answer is that the goal is to use philosophical analysis to distill a core, unifying concept that will cover all cases. However, as above, there are many who worry this project is not achieveable. Another answer is as follows: the goal is to describe the range of different concepts at play in cognitive science, and account for their explanatory purchase. On this view, the business of licensing content needs to be sensitive to the variety of representational concepts at play in cognitive science. Pluralism, then, looks unavoidable.

Recent work by Nick Shea builds on this idea. Shea’s approach is to look at the way cognitive scientists use notions of representation to successfully explain behaviour. The result of this process is a “varitel” semantics, which combines teleosemantics and structural correspondence (Shea, 2018, Chapter 2). Both offer organisms a relation with external circumstances that they are able to exploit. On Shea’s view, pluralism is a commitment of this explanatory strategy:

We may get one theory of content that gives us a good account of the correctness conditions involved in animal signalling, say, and another one for cognitive maps in the rat hippocampus. There is no need to find a single account that covers both.

Shea (2018, p. 43)

For both Godfrey-Smith and Shea, exploring pluralist strategies offers the best way forward for those attempting to produce naturalised theories of content. Our account is developed with this general methodological commitment in view. But why is building in an etiological account of the content of signals in generative hierarchies useful? Our answer to this question is that there are, and are likely to be, many cases where doing so can help account for explanatory success in cognitive science. And if predictive processing—as a general theory of cognition—is to be applied to these cases, then building in teleosemantics is an important project. Covering the range of cases that might require teleosemantic treatment is well beyond the scope of this paper. However, below we run through a brief case study in order to illustrate the thinking behind it.

### Practice-Oriented Pluralism and Predictive Processing

As we have outlined, on Nick Shea’s view philosophical theories of content should be guided by cases of explanatory success in the cognitive sciences (Shea, 2018). And, given that cognitive science deals with such a broad range of cases, it is unsurprising that this process will produce a range of different approaches to content. Here we briefly run through an illustrative case: that of decision making in Rhesus monkeys. However, it is worth noting that Shea offers a wide variety of cases, from neural network models (Shea, 2018, Section 4.3) to animal signalling (Shea, 2018, Section 4.5). It is also important to note what is being claimed by Shea (and ourselves) in these cases. The claim is not that no other account of content might be capable of explaining the results produced in these studies. Rather the claim is that, when we look to these studies, we find that the type of content used to do the explanatory work is best captured by teleosemantics. To put this another way, the question is not “which theory of content best covers all these cases?", it is “which theory best accounts for explanatory success in this particular experimental case?". This reflects the practice-oriented approach: the role of philosophy is to describe the representational concepts that are being employed in successful scientific practice.

Teleosemantics is an *outcome-oriented* theory of content. Shea incorporates this notion into his theory of function, using the term *consequence etiology* (Shea, 2018, p. 48). Roughly the idea is that certain processes, such as natural selection and learning, stabilise traits in an organism. Shea’s account of function differs from the notion of proper function we’ve been working with, and the magnitude of that difference depends on the use to which the notions are put. One thing they have in common is that they fit naturally with studies employing reward-based learning paradigms, in particular the research cluster around the neurophysiology of reward. Many studies in this area aim to identify the values and likelihoods of reward functions, where those values represent external circumstances that are good or bad outcomes for the experimental subject. Behaviour stabilises in a subject—such as our lever-pushing rat—because certain signals in the subject’s cognitive system start to reliably correlate with specific rewards. In the opening paragraph of his overview on the neurophysiology of reward paradigm, Wolfram Schultz writes:

The functions of rewards are based primarily on their effects on behavior and are less directly governed by the physics and chemistry of input events as in sensory systems. Therefore, the investigation of neural mechanisms underlying reward functions requires behavioral theories that can conceptualize the different effects of rewards on behavior. The scientific investigation of behavioral processes by animal learning theory and economic utility theory has produced a theoretical framework that can help to elucidate the neural correlates for reward functions in learning, goal-directed approach behavior, and decision making under uncertainty.

Schultz (2006, p. 87)

It is easy to see why teleosemantics is well-placed to “conceptualize the different effects of reward on behaviour", and more why this research program aligns well with a consequence etiology account of function. It gives us a precise way of showing how learning processes in a system can come to represent the utility of beneficial external outcomes.

For instance, in a study presented by Kiani and Shadlen (2009), Rhesus monkeys were given a post-decision wagering task. Subjects were required to make decisions about the overall direction of motion in a dynamic random dot display. The difficulty of this task was specified by the percentage of coherently moving dots and the length of time the display was viewed for. Saccadic eye movement was used to identify the monkey’s decision, directed toward either a right or left visual target. Correct decisions were given a liquid reward, while incorrect decisions were not. Finally, the monkeys were given a “sure target"; that is, a target in the centre of the screen that guaranteed a reward, but at approximately 80% of the liquid reward for a correct choice. The thought was that the monkeys would opt for the sure target as the difficulty of the task went up, which in turn would reflect the level of certainty they had in their ability to successfully complete the initial task. Kiana and Shadlen’s results supported this hypothesis.

Now, suppose we want to understand this experimental data using a predictive processing framework. We need some way of understanding how the value of an external success-condition (the reward) comes to be represented by internal mechanisms, such that we can explain the behaviour of the subjects, and in particular the way the uncertainty and reward values are balanced. As a teleosemantic treatment of internal signals gives us a consequence etiology account of function, it is well placed to deliver on this explanatory task. More broadly, this shows that, if we adopt the practice-oriented approach, developing a range of theories of content for predictive processing systems is an important task. This is because it gives us the tools to explain the broad range of experimental paradigms and results we find across the cognitive sciences.

## Conclusion

Our goals in this paper were twofold. First, we wanted to show how a teleosemantic account of content for prediction and prediction error signals could mesh with a broader causal-probabilistic account of generative heirarchies. We argued this process revealed important similarities between the explanatory motivations and conceptual machinery employed by teleosemantics and predictive processing. Second, we wanted to advocate the virtues of pluralist approaches to representational content. We followed Peter Godfrey-Smith and Nick Shea in maintaining that a single, overarching account of content for cognitive science is unlikely to be successful. Cognitive scientists employ a range of different content-invoking concepts, and philosophers should be developing frameworks that respect this theoretical diversity. We think this is a good reason to issue predictive processing with a pluralist licence for content.

## Footnotes

1 How much attention cognitive science departments pay to this licensing system varies by department, but at least some appear to take it seriously.

2 There are those who deny that predictive processing should be understood in representationalist terms; e.g. Hutto (2018). Here we sideline such debates. Our aim is to provide a teleosemantic analysis of signals in predictive processing systems for those who want to understand such systems in representational terms.

3 We largely follow the program outlined by Nick Shea here (Shea, 2018, Sections 2.2 and 2.6).

4 Notably, we do not go into the role of precision estimates in prediction error minimisation. This is for reasons of space, and because we do not think such detail affects our argument.

5 There are some who believe predictive processing offers a general theory of the brain, encompassing all our mental processes e.g. Hohwy (2013). Others are more cautious (Clark, 2013, §5.2). We are sceptical of the more ambitious formulations of predictive processing. All we rely on here is the claim that prediction error minimisation governs perception and action.

6 Other contentful aspects of the mechanism, such as the hypotheses contained in models, are accounted for via structural resemblance. We go into more detail in the following section.

7 ‘Likelihood’ is a technical term for a conditional probability that plays a specific role in Bayesian inference. Somewhat confusingly, the likelihood of an *hypothesis* with respect to some data is the conditional probability of that *data* given that hypothesis.

8 Here and throughout we refer to Millikan’s (2004, §6) teleosemantic theory. For a fuller exposition of this version of teleosemantics see Mann & Pain (2022).

9 As noted in Mann & Pain (2022), the question of the relationship between different processes of selection, especially between learning and natural selection, has been much discussed and is not settled (Artiga, 2010; Baigrie, 1989; Catania, 1999; Hull et al., 2001; Kingsbury, 2008; Skinner, 1981; Watson & Szathmáry, 2016). Teleosemantics requires that there be an explanatorily relevant similarity in the processes that give rise to functional behaviours. Millikan (1984, §§1–2) defends this claim extensively in defining proper function.

10 More precisely: the content of a signal picks out which state would have to obtain in order for the signal to bear the appropriate relation to it. The appropriate relation is the one that must hold for the consumer’s response to successfully achieve the effect.

11 Here we follow Wiese (2017) in treating action as being prompted by systematic misrepresentation. On an alternative view, the descriptive content of the prediction is not that the lever is *currently* being pressed (the falsity of which prompts action) but that the lever *will* be pressed in the near future (the truth of which is ensured by action) (Smith et al., 2022). Since we’re telling the story in terms of misrepresentation, we might be subject to a broader set of issues that have been raised for teleosemantics in the past. We leave open whether these problems, if they arise, should be confronted directly, or whether the appropriate response is to switch to the ‘true prediction’ account. Thanks to an anonymous reviewer for raising this point.

12 Shea uses the term “actor-critic model”. In this sense a model is a scientific device used to represent brain activity. In the sense we have been employing the term, a model is a component in a generative hierarchy that represents causal-probabilistic features of the world. To avoid confusion, in this section we will use the term ‘framework’ to label scientific models.

13 This illustrates an important point. According to predictive processing, the primary goal of cognitive systems is to minimise error. In general, this will produce the result that, over the long-term, models will become more accurate with respect to the world. But this will not always be the case. Sometimes the impetus to minimise error early in the hierarchy will lead to inaccuracies between models and the world. Illusory cases such as these draw a lot of interest from proponents of the view, as error minimisation is thought to provide an explanation for such perceptual phenomena. See for instance Hohwy’s discussions of binocular rivalry or the rubber hand illusion (Hohwy, 2013).

14 This is not to say that structural representation accounts *do not* mesh with predictive processing systems. Rather, we are simply motivating the thought that teleosemantics is likewise a good fit.

15 Thanks to an anonymous reviewer for raising these.

16 In not one but two of Agatha Christie’s *Poirot* mysteries (‘The Disappearance of Mr Davenheim’ and ‘The Mystery of Hunter’s Lodge’) the eponymous detective solves the crime without leaving his home, relying solely on information provided by Inspector Japp and Captain Hastings—neither of whom figures out the solution before Poirot reveals it.

17 This is not to suggest that these theories are uniquely problematic. In this respect, they are in exactly the same position as every philosophical theory.
